# APAatlas: decoding alternative polyadenylation across human tissues

**DOI:** 10.1093/nar/gkz876

**Published:** 2019-10-05

**Authors:** Wei Hong, Hang Ruan, Zhao Zhang, Youqiong Ye, Yaoming Liu, Shengli Li, Ying Jing, Huiwen Zhang, Lixia Diao, Han Liang, Leng Han

**Affiliations:** 1 Department of Biochemistry and Molecular Biology, McGovern Medical School at The University of Texas Health Science Center at Houston, Houston, TX 77030, USA; 2 Department of Bioinformatics and Computational Biology, The University of Texas MD Anderson Cancer Center, Houston, TX 77030, USA; 3 Center for Precision Health, The University of Texas Health Science Center at Houston, Houston, TX 77030, USA

## Abstract

Alternative polyadenylation (APA) is an RNA-processing mechanism on the 3′ terminus that generates distinct isoforms of mRNAs and/or other RNA polymerase II transcripts with different 3′UTR lengths. Widespread APA affects post-transcriptional gene regulation in mRNA translation, stability, and localization, and exhibits strong tissue specificity. However, no existing database provides comprehensive information about APA events in a large number of human normal tissues. Using the RNA-seq data from the Genotype-Tissue Expression project, we systematically identified APA events from 9475 samples across 53 human tissues and examined their associations with multiple traits and gene expression across tissues. We further developed APAatlas, a user-friendly database (https://hanlab.uth.edu/apa/) for searching, browsing and downloading related information. APAatlas will help the biomedical research community elucidate the functions and mechanisms of APA events in human tissues.

## INTRODUCTION

The maturation of 3′ ends of mRNA and/or other RNA polymerase II products (i.e. long-noncoding RNA) involves cleavage at the poly(A) site (PAS) and synthesis of a poly(A) tail. Alternative polyadenylation (APA) is a phenomenon in which multiple PASs of the same gene are recognized by cleavage and polyadenylation machinery, leading to transcript isoforms with various 3′ untranslated regions (3′-UTRs), which may further regulate mRNA translation, stability and localization (1). APA is conserved and common among eukaryotes (1) and can be found in >70% of human genes (2). Dynamic APA changes occur during cell proliferation and differentiation and exhibit strong tissue specificity (1,3–5). For example, the testis as well as proliferating CD4^+^ T cells tend to have proximal PASs (5,6), while brain and neuronal cells tend to have longer 3′-UTRs (6,7). Growing evidence suggests that APA participates in human diseases, especially in cancer (8–11).

Early APA databases (i.e. PolyA_DB2 (12) and PACdb (13)) were based on expressed sequence tags, which could only detect limited APA events. The fast development of next-generation sequencing has provided opportunities for detecting genome-wide APA events. Several APA databases, including APADB (14), APASdb (15) and PolyA_DB3 (16), were developed based on 3′-sequencing data, but collected only small amounts of human tissues and disease samples. In contrast, RNA-seq has become the standard technology for transcriptome profiling and has been applied in large-scale genomic studies. Several algorithms have been developed for identifying APA events from RNA-seq data (17), either based on *de novo* identification algorithms (i.e. DaPars (9) and IsoSCM (18)) or annotation-based algorithms (i.e. QAPA (19) and SAAP-RS (7)). TC3A provided comprehensive compilation of APA events in thousands of tumors from The Cancer Genome Atlas (20). However, no existing database has collected APA events in a large number of human normal tissues.

The Genotype-Tissue Expression (GTEx) project provides, the largest collection of RNA-seq data in >50 human normal tissues, along with numerous traits, and thus can be a good resource for studying gene expression and other RNA changes in normal tissues (21). Based on GTEx data, we characterized APA events across 53 human normal tissues and developed the APAatlas, a database we have used to explore the APA landscape across tissues and investigate the relevance between APA usage and GTEx traits or gene expression.

## DATA COLLECTION AND PROCESSING

### RNA-seq data collection and processing

We downloaded 9,611 GTEx RNA-seq files across 548 human donors from the database of Genotypes and Phenotypes (dbGaP) (22) (https://www.ncbi.nlm.nih.gov/gap/) under accession number phs000424.v7.p2 on 26 July 2018 in sra format and converted them into standard fastq using the fastq-dump program of the SRA toolkit (23) (https://trace.ncbi.nlm.nih.gov/Traces/sra/). For all replicates (the same sample with two or more RNA-seq files), we retained only the file with the largest number of reads (24). Thus, we retained 9475 RNAseq sample files, encompassing 53 normal tissues. We aligned RNA-seq reads to the human genome (hg19/GRCh37) using HISAT2 (version 2.1.0) (25). We downloaded the expression matrix (release v7) and trait datasets (pht002742.v7.p2) from the GTEx portal (https://www.gtexportal.org/home/) and dbGaP, respectively, on 08/10/2018. We collected gene annotations from GENCODE v19 (26) (https://www.gencodegenes.org/) and RefSeq (27) (https://www.ncbi.nlm.nih.gov/refseq/).

### Quantification of alternative polyadenylation

**Figure 1.**
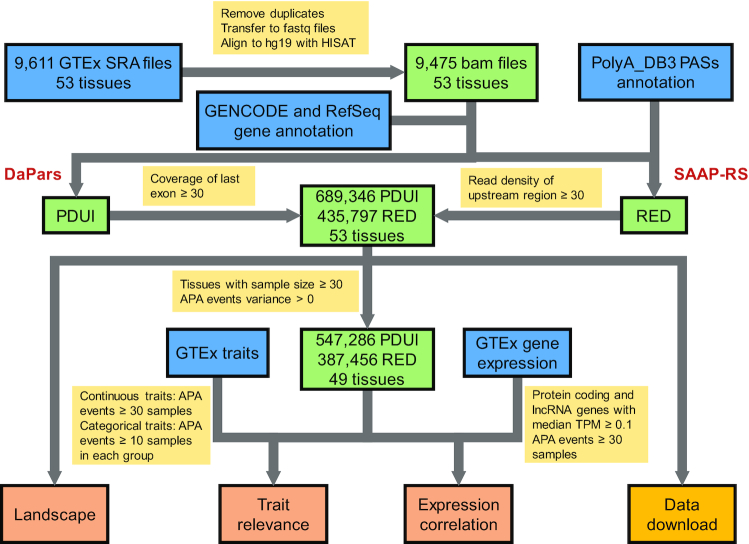
Construction pipeline of the APAatlas database. RNA-seq raw data, gene expression and traits involved in 53 human tissues were collected from the Genotype-Tissue Expression (GTEx) portal. Two algorithms were used to quantify alternative polyadenylation (APA) usage: The percentage of distal poly(A) site usage index (PDUI) was *de novo* identified by DaPars; the relative expression difference (RED) was quantified by the annotation-based algorithm SAAP-RS and further filtered by sample size and APA events for subsequent analysis. Users can query the APA landscape, trait relevance, and gene expression correlation in three modules and download APA usage matrixes.

While annotation-based algorithms can benefit from 3′-sequencing-based APA site annotations, recent studies have also demonstrated the possibility of *de novo* identification algorithms to identify novel APA events in a large number of samples (9,10,20). Therefore, we utilized two popular algorithms to quantify APA usage from standard RNA-seq data (Figure 1). We first used the well-established *de novo* algorithm DaPars with RefSeq hg19 annotation (9) to identify the alternative proximal PAS within each tissue. Based on the two-PAS model, DaPars applies a linear regression model to infer the location of the APA site within the 3′ UTR region. We quantified the relative abundance of long and short 3′ UTR isoforms as the percentage of the distal poly(A) site usage index (PDUI). We discarded the PDUIs of certain transcripts for which the coverage of the last exon <30× , as previously described (9). We retained transcripts that had non-missing values in at least one sample.

We also applied an annotation-based algorithm, the significance analysis of alternative polyadenylation using RNA-seq (SAAP-RS), (7) on the GTEx RNA-seq data. We downloaded the annotations of the PASs from PolyA_DB3 (16). For each gene in GENCODE v19 annotation, we kept the longest transcript (16) and chose the first conserved PAS located in the 3′-most exon as the reference PAS (7). We defined the upstream region as being from the stop codon to the reference PAS and the downstream region as being from the reference PAS to the last PAS. For each gene, we quantified APA usage using the relative expression difference (RED), log2(DN/UP), where DN and UP are the number of reads respectively mapped into the downstream and upstream regions. SAAP-RS required the read density (read number/region length) of the downstream region to be lower than that of the upstream region (7), and we further required the read density of the upstream region to be ≥ 30. We retained transcripts with a detected RED in at least one sample. We then normalized the RED for each gene by standardization (subtracted the mean and divided by the standard deviation) across all 9,475 samples.

## DATABASE CONTENT AND USAGE

### APA landscape across tissues

In total, we identified 689 346 APA events (median: 13 063 per tissue) with DaPars and 435,797 APA events (median: 8322 per tissue) with SAAP-RS (Supplementary Table S1). DaPars has identified significantly more APA events than SAAP-RS (Supplementary Figure S1). Because *de novo* identification may introduce false positives, we utilize two methods in our data portal. We recapitulated previously published findings with regard to tissue-specific APA. For example, we observed that brain tissue tends to harbor longer 3′UTR isoforms, whereas blood and testicular tissues tend to have shorter 3′UTRs (Supplementary Figure S2). We also found that several genes had greater tendency to have long 3′UTR isoforms in brain tissues than in the testis and liver (Supplementary Figure S3), which is consistent with the findings from previous studies (3–5). To ensure adequate statistical power, we filtered the APA events with zero variance and performed further analysis for tissues with sample size ≥30 (Figure 1). For each gene, we used the Wilcoxon test (two tissues) or Kruskal–Wallis test (three or more tissues) to test the significant difference in APA usage among tissues.

### Association between APA and traits

For continuous traits (age, height, weight and body mass index), we used Spearman's rank correlation to calculate the correlation coefficient (*Rs*), with APA events ≥ 30 samples in a tissue (Figure 1). We defined significant correlation as the absolute value of *Rs* > 0.3 and false discovery rate (FDR) <0.05. For categorical traits (sex, race, autolysis score, hardy scale and ischemic time), we used the Wilcoxon test (two groups) or Kruskal-Wallis test (three or more groups) to test the significant difference in APA usage among trait groups with ≥ 10 samples (Figure 1). We defined a significant difference across different groups in categorical traits as FDR <0.05. In total, we identified 107 305 (PDUI) and 91 687 (RED) APA events relevant to at least one trait (Supplementary Table S1).

### Correlation between APA and gene expression

Protein coding genes and lncRNA genes with median transcripts per million ≥ 0.1 in a tissue remained for analysis. We used Spearman's rank correlation to calculate the correlation coefficient (*Rs*) between APA and gene expression and performed multiple correction by FDR. We defined significant correlation between the gene expression level and APA usage as the absolute value of *Rs* >0.3 and FDR <0.05. In total, 285 083 (PDUI) and 234 092 (RED) APA events were significantly correlated with the expression of at least one gene (Supplementary Table S1).

### Web design and interface

**Figure 2.**
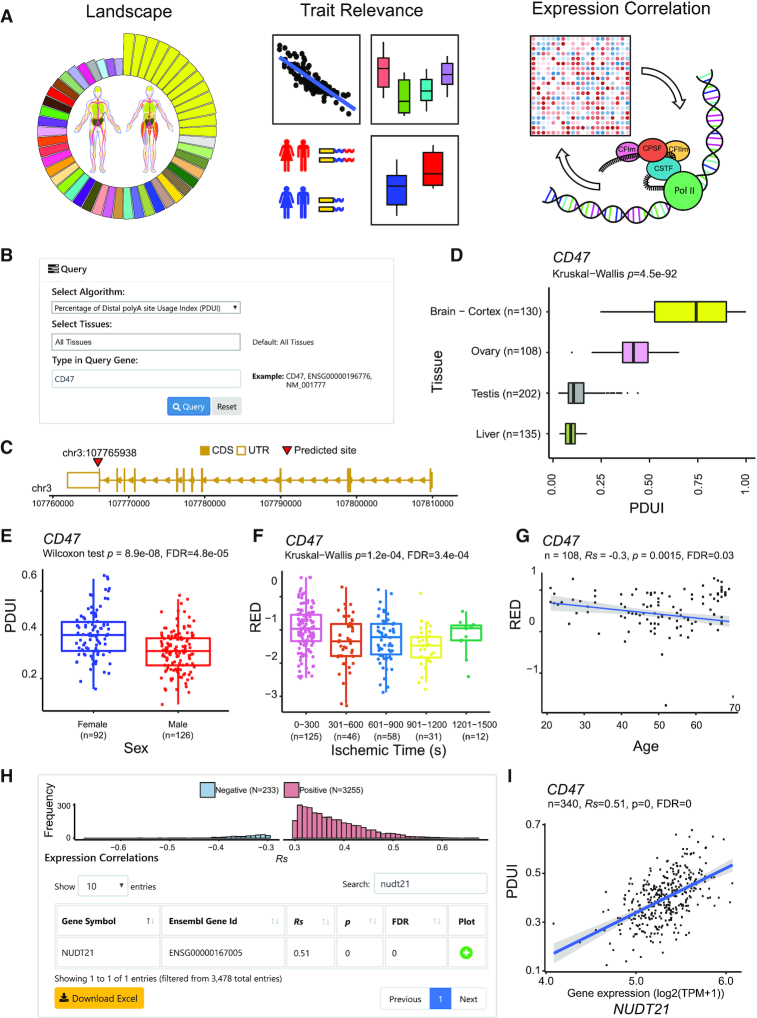
Overview of APAatlas database. (**A**) Three modules of APAatlas, including landscape, trait relevance, and expression correlation. (**B**) Query interface of landscape module. Users can select the algorithm and tissue(s), search APA events by gene name, Ensembl gene id or RefSeq transcript ID. (**C**) DaPars located the APA site of *CD47* at chr3:107,765 938, in the 3′-UTR of the last exon. (**D**) Example of the APA usage landscape across tissues: PDUI of gene *CD47* significantly differed among the brain cortex, ovary, testis and liver. (E–G) Examples of APA events relevant to traits and gene expression: (**E**) in breast tissue, PDUI of *CD47* showed a sex difference; (**F**) in whole blood, RED of gene *CD47* differed among ischemic groups; (**G**) RED of gene *CD47* negatively correlated with age in ovarian tissue. (**H**) Example of gene expression correlated with PDUI of *CD47* in subcutaneous adipose tissue. Histogram showing the distribution of positively and negatively correlated genes; table showing the information of one correlated gene *NUDT21*. (**I**) In subcutaneous adipose tissue, PDUI of *CD47* positively correlated with the expression of gene *NUDT21*.

APAatlas provides an easily accessible web interface developed in Bootstrap 4, a JavaScript framework that includes HTML, CSS and JavaScript code designed for creating responsive websites and web applications (http://getbootstrap.com/). The website was developed using Python (2.7.2), based on the Django web-framework. Data querying, retrieval and plotting were performed by R (3.5.3). Three modules are provided for users to view the APA landscape across tissues, trait relevance, and expression correlation of certain APA events (Figure 2A). In each module, users can select algorithms and tissues and query APA events by typing the gene symbol, RefSeq transcript ID or Ensembl gene ID in the search box (Figure 2B). The search box is case- and space- insensitive.

### Data browsing and querying of three modules

Querying on the ‘Landscape’ page returns a table with an algorithm, gene symbol, RefSeq transcript ID, and the Ensembl gene ID (28) of the queried gene name. For each record, a gene structure plot is provided to display the exact coordinate of the APA site, and a diagram of the boxplot is provided to display the APA landscape across selected tissues. For example, DaPars located the APA site of gene *CD47* at chr3:107 765 938 in the 3′-UTR of the last exon (Figure 2C). The PDUI of gene *CD47* is significantly different among tissues from the brain cortex, ovary, testis and liver (Figure 2D).

Querying on the ‘Trait Relevance’ page, users can select the trait(s) of interest. Details with the algorithm, tissue, gene symbol, RefSeq transcript ID, Ensembl gene ID *P*-value, sample size and significance (*Rs* and FDR for continuous traits; FDR for categorical traits) will be displayed. A diagram of the boxplot (for categorical traits) or Spearman's correlation dot plot (for continuous traits) is embedded in each trait of each record to display the association between APA events and traits. For example, the PDUI of gene *CD47* showed sex differences in breast tissue (Figure 2E), while the RED of *CD47* differed among ischemic groups in whole blood (Figure 2F) and correlated with age in ovarian tissue (Figure 2G). Users can also view the gene structure and APA site by clicking the ‘APA Site’ button.

Querying on the ‘Expression Correlation’ page will return the algorithm, gene symbol, RefSeq transcript ID, Ensembl gene ID as well as the gene structure plot of the APA events. For each APA event, a table containing the gene symbol, Ensembl gene ID, *Rs*, *P*-value and FDR of each significantly expression-correlated gene can be retrieved by clicking the ‘Details’ button (Figure 2H). Users can further retrieve the Spearman's correlation dot plot for each correlated gene. For example, in subcutaneous adipose tissue, the PDUI of gene *CD47* is positively correlated with the expression of gene *NUDT21*, which is a key APA regulator (29) (Figure 2I). If the number of significantly correlated genes ≥ 10, a histogram of the distribution of positively and negatively correlated genes is provided (Figure 2H).

The sample size and number of APA events of each tissue are displayed in the ‘Dataset’ page. APAatlas also provides a detailed ‘Documents’ page describing how to search and browse APA events, as well as data collection and analytic methods.

### Download of data, figures and tables

All the plots generated in the three modules can be downloaded in PDF format by clicking the ‘Download PDF’ button below the plot. A trait relevance table and gene expression correlation table can be downloaded in Excel format by clicking the ‘Download Excel’ link below the table. On the ‘Downloads’ page, users can download the full APA usage matrix through the download links or query the APA usage matrix by tissue type. In addition, we designed a ‘Download Data’ button in the ‘Landscape’ module for users to download the APA usage data of single genes in select tissues. Matrix files are in plain text and compressed into zip format. In each matrix, APA events are indexed by rows. The first three columns represent the gene symbol, RefSeq transcript ID and Ensembl gene ID; the remaining columns represent PDUI or RED values for each sample.

## SUMMARY AND FUTURE DIRECTIONS

We developed the APAatlas as a user-friendly database for querying and downloading APA usage in human tissues. By utilizing the large-scale RNA-seq datasets from GTEx, APAatlas provides the most comprehensive APA usage for 9475 samples across 53 human tissues. In APAatlas, we identified 689 346 APA events with PDUI and 435 797 APA events with RED. APAatlas further provides specialized modules for users to query the APA landscape across tissues, trait relevance, and correlations between APA usage and gene expression. We only considered APA events in annotated 3′-UTR regions with the two-PAS model utilized by these two algorithms (7,9). We will update the APAatlas to include more human samples and will maintain it as a useful resource for the research community. APA is the emerging mechanism involved in the regulation of mRNA translation, stability and localization. It will be necessary to further investigate the regulating mechanisms of APAs through integrative analysis.

## Supplementary Material

gkz876_Supplemental_File
