# Supplementary material for: APAatlas: decoding alternative polyadenylation across human tissues
Source: Nucleic Acids Res. 2019 Oct 5;48(D1):D34–9. doi: 10.1093/nar/gkz876 (PMC6943053; doi:10.1093/nar/gkz876)
Supplement: gkz876_Supplemental_File [file gkz876_supplemental_file.pdf]

SUPPLEMENTARY FIGURES

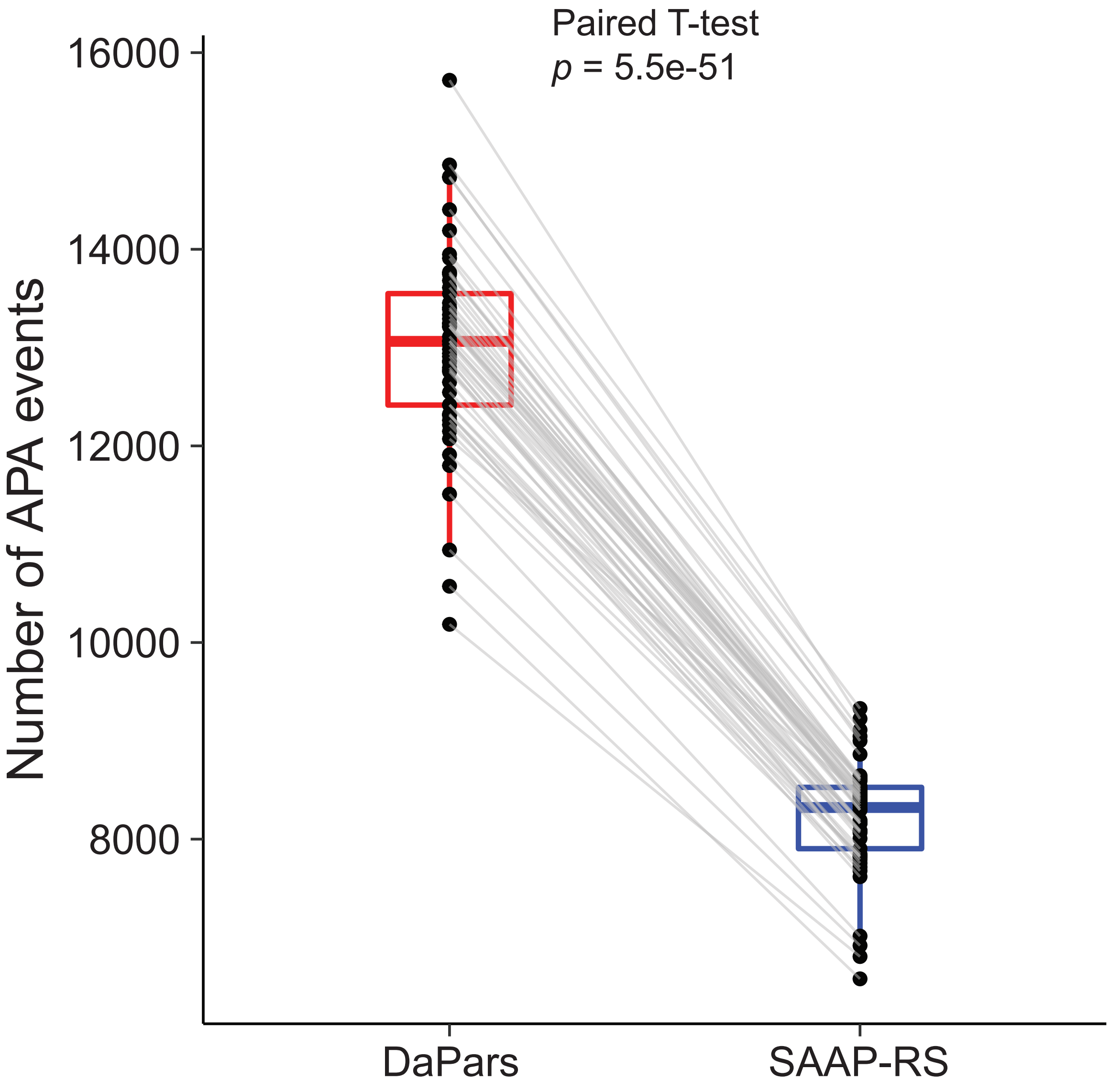

Figure S1. Comparison of numbers of APA events identified by DaPars and SAAP-RS.

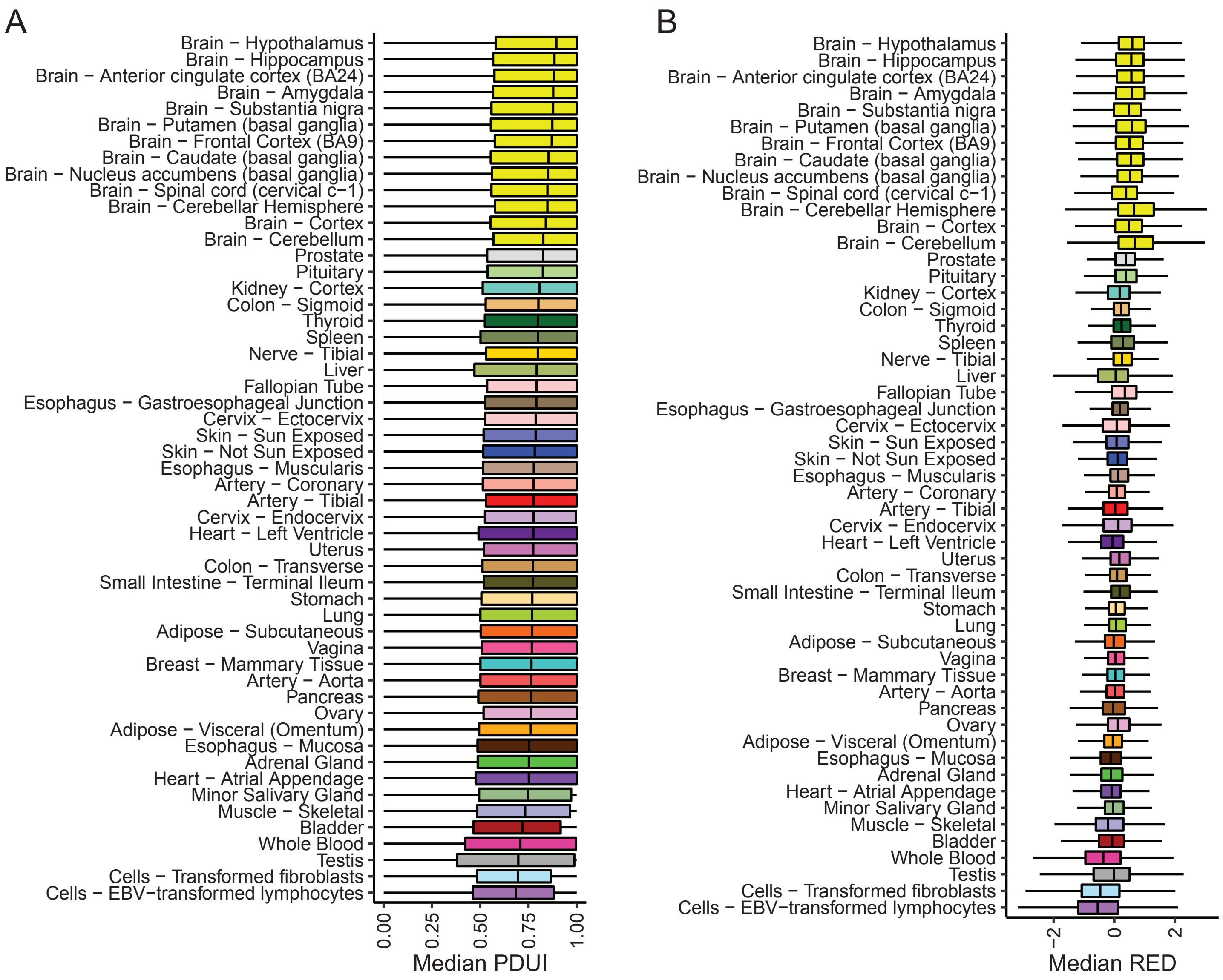

Figure S2. Overall (A) PDUI and (B) RED landscape in 53 tissues. Tissues were sorted from top to bottom in descending order of median PDUI or RED values.

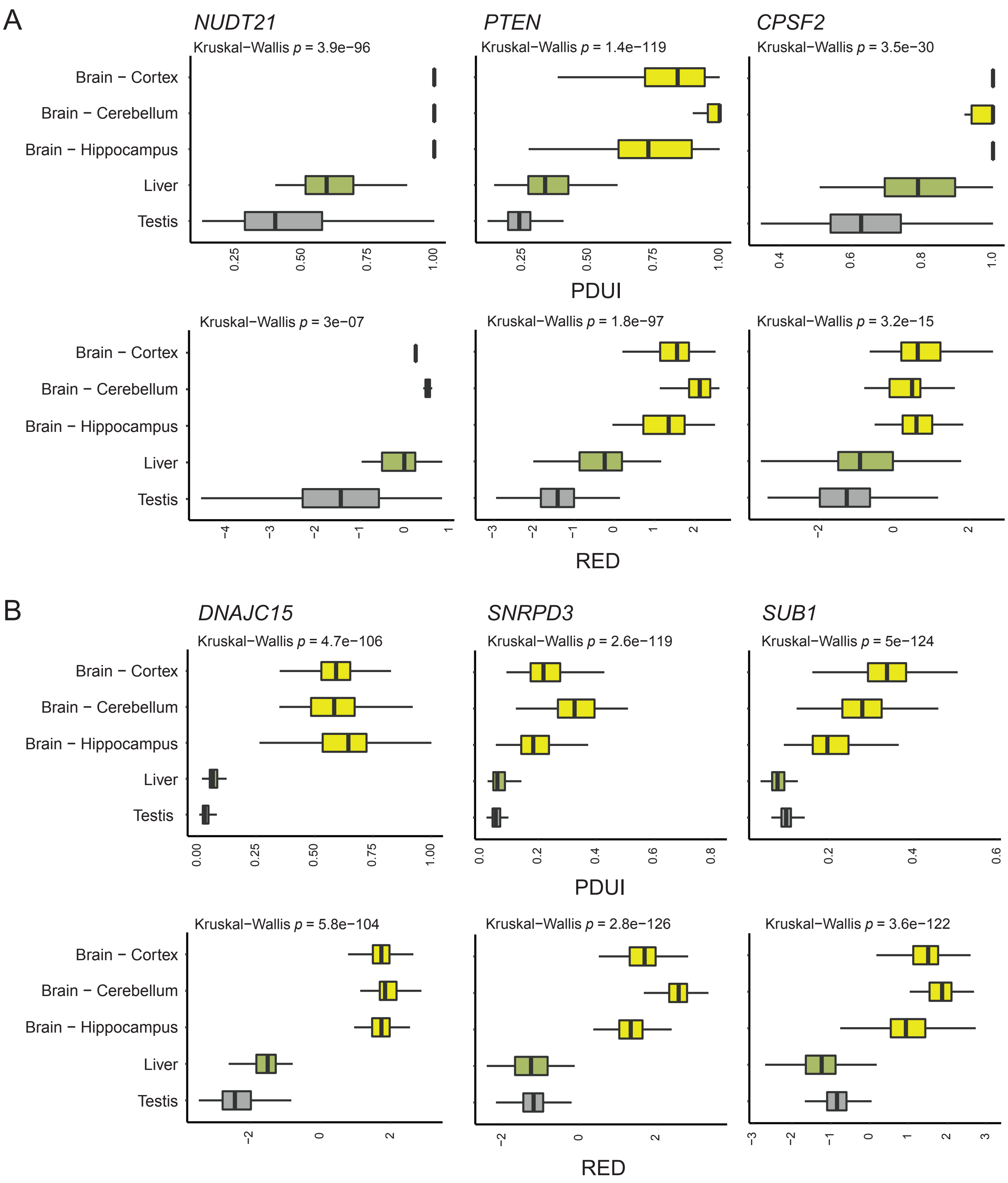

Figure S3. APA usage of six genes in tissues from the brain, liver, and testis. (A) *NUDT21*, *PTEN* and *CPSF2* have greater tendency to have long 3'-UTR in the brain cortex and cerebellum compared with the testis. (B) *DNAJC15*, *SNRPD3* and *SUB1* have greater tendency to have long 3'-UTR in the brain hippocampus compared with the liver.

SUPPLEMENTARY TABLE

Table S1 Summary of APA events for each tissue in APAatlas.

Tissues	Sample size	No. of APA (PDUI)			No. of APA (RED)		
		Total	Relevant with traits	Correlated with expression	Total	Relevant with traits	Correlated with expression
**Adipose - Subcutaneous**	385	14403	1547	6252	9046	2007	5496
**Adipose - Visceral (Omentum)**	235	13210	5503	7062	8418	4156	5743
**Adrenal Gland**	161	12545	5685	7209	8166	3576	5338
**Artery - Aorta**	248	12419	3583	6643	8078	3378	5390
**Artery - Coronary**	141	12940	3520	6265	8474	2798	5114
**Artery - Tibial**	361	13948	631	6714	8863	1279	5497
**Bladder**	12	10185	NA	NA	6806	NA	NA
**Brain - Amygdala**	83	12325	280	3410	7678	477	3184
**Brain - Anterior cingulate cortex (BA24)**	100	12754	601	4982	8089	671	4125
**Brain - Caudate (basal ganglia)**	135	13214	29	5149	8160	189	4322
**Brain - Cerebellar Hemisphere**	120	14190	519	6772	8616	791	4897
**Brain - Cerebellum**	147	12415	223	5672	7903	756	4553
**Brain - Cortex**	133	12866	525	4663	7756	906	3867
**Brain - Frontal Cortex (BA9)**	122	13758	530	5756	8527	718	4540
**Brain - Hippocampus**	104	12852	67	4347	8007	316	3927
**Brain - Hypothalamus**	104	13610	677	5251	8510	794	4351
**Brain - Nucleus accumbens (basal ganglia)**	125	13033	10	5848	8322	322	4548
**Brain - Putamen (basal ganglia)**	105	12148	28	3971	7829	141	3583
**Brain - Spinal cord (cervical c-1)**	76	12314	1	3525	7819	6	3313
**Brain - Substantia nigra**	72	12796	1	3682	7849	34	3313
**Breast - Mammary Tissue**	221	13744	1734	6158	8646	2504	5451
**Cells - EBV-transformed lymphocytes**	138	12313	23	7460	8192	22	5298
**Cells - Transformed fibroblasts**	305	12071	83	7710	8455	51	6005
**Cervix - Ectocervix**	6	10941	NA	NA	6918	NA	NA
**Cervix - Endocervix**	5	10572	NA	NA	6579	NA	NA
**Colon - Sigmoid**	175	13334	5859	6829	8406	3986	5339
**Colon - Transverse**	207	13406	3875	6511	8442	3244	5426
**Esophagus - Gastroesophageal Junction**	177	13387	5390	6640	8499	3771	5148
**Esophagus - Mucosa**	338	13454	7424	8004	8592	4984	5970
**Esophagus - Muscularis**	288	13214	6440	7434	8635	4318	5669
**Fallopian Tube**	7	11509	NA	NA	7014	NA	NA
**Heart - Atrial Appendage**	219	12215	5557	6721	8066	3715	5053
**Heart - Left Ventricle**	274	13063	5866	6671	8483	3893	4842
**Kidney - Cortex**	38	12648	859	1970	7792	416	1890

**Liver**	141	11910	4977	5542	7827	3215	4321
**Lung**	379	14858	4728	8114	9329	4110	6301
**Minor Salivary Gland**	71	12982	3	2968	8084	3	3143
**Muscle - Skeletal**	477	13112	2386	5438	8615	2678	4616
**Nerve - Tibial**	328	13768	779	6989	8608	1529	5828
**Ovary**	112	12909	2538	5504	8182	2112	4725
**Pancreas**	203	13293	720	3913	8538	925	3526
**Pituitary**	126	13908	0	5562	8377	69	5008
**Prostate**	120	13682	2750	5495	8351	2399	4982
**Skin - Not Sun Exposed**	270	13240	2038	6690	8395	2886	5604
**Skin - Sun Exposed**	400	14730	1647	6930	9111	1984	5672
**Small Intestine - Terminal Ileum**	106	13549	26	4659	8297	24	4368
**Spleen**	121	12260	23	5103	7618	27	4613
**Stomach**	209	13068	1906	5608	8297	1697	5003
**Testis**	207	15719	3595	8547	9224	2890	6061
**Thyroid**	365	14739	4646	7121	9000	4268	5933
**Uterus**	93	12772	3946	5910	8160	3087	4899
**Vagina**	99	13251	1118	5149	8433	1098	4560
**Whole Blood**	281	11800	2409	4560	7716	2467	3737
